# Evaluation of the Water Sorption and Solubility Behavior of Different Polymeric Luting Materials

**DOI:** 10.3390/polym13172851

**Published:** 2021-08-25

**Authors:** Nawaf Labban, Rasha AlSheikh, Melvin Lund, Bruce A. Matis, B. Keith Moore, Michael A. Cochran, Jeffrey A. Platt

**Affiliations:** 1 Department of Prosthetic Dental Sciences, College of Dentistry, King Saud University, Riyadh 11545, Saudi Arabia; 2 Department of Restorative Dental Science, College of Dentistry, Imam Abdulrahan Bin Faisal University, Dammam 34212, Saudi Arabia; ralsheikh@iau.edu.sa; 3 Department of Restorative Dentistry, Indiana University School of Dentistry, Indianapolis, IN 46202, USA; melvin.lund@comcast.net (M.L.); bmatis@iu.edu (B.A.M.); ralsheik@gmail.com (B.K.M.); mcochran@iu.edu (M.A.C.); 4 Department of Biomedical Sciences and Comprehensive Care, Division of Dental Materials, Indiana University School of Dentistry, Indianapolis, IN 46202, USA; jplatt2@iu.edu

**Keywords:** polymeric cements, water solubility, water sorption, luting agents, self-adhesive

## Abstract

*Objective*: The study evaluated the water sorption (WSP) and water solubility (WSL) characteristics of different luting agents over a 180-day water storage period. *Materials and Methods*: Nine luting materials, i.e., conventional resin cement: Panavia F (PF), Rely X ARC (RA), self-adhesive resin cement: Rely X Unicem (RU), Breez (BZ), Maxcem Elite (MX), BisCem (BC) and resin-modified glass ionomer cement: FujiCem (FC), FujiPlus (FP) Rely X luting plus (RL) were assessed and fifty-two-disc specimens of each material were fabricated. All specimens were desiccated until a constant weight (W0) was reached. Thirteen specimens for each luting material were then randomly assigned to one of the four water immersion periods (7, 30, 90, and 180 days). After each period, the specimens were removed from the water and weighed to get W1. The samples were again desiccated for a second time and W_2_ was measured. Both WSP and WSL were determined by the following equations: WSP (%) = (W_1_ − W_2_) × 100/W_0_ and WSL (%) = (W_0_ − W_2_) × 100/W_0_. Assessments were performed following ISO standards. ANOVA was used to assess the effect of luting agent and time period on water sorption and solubility. Pair-wise comparisons were adjusted using Tukey’s multiple comparison procedure. A significance level of 0.05 was used for all statistical tests. *Results*: The highest mean WSP and WSL (WSP/WSL) were demonstrated by resin-modified glass-ionomers (RL 18.32/3.25, FC 17.08/4.83, and FP 14.14/1.99), while resin luting agents showed lower WSP and WSL results (PF 1.6/0.67 and RA 1.76/0.46), respectively. The self-adhesive agents exhibited a wide range of WSP and WSL values (RU 1.86/0.13, BZ 4.66/0.93, and MX 3.68/1.11). Self-adhesive cement showed lower WSP and WSL compared with the resin-modified glass-ionomers (*p* < 0.05). All the materials reached equilibrium after 90-days. *Conclusions*: Resin-based luting materials have the lowest sorption and solubility. Rely X Unicem self-adhesive luting materials were comparable to resin luting materials for WSL and WSP. Resin-modified glass-ionomer showed the highest water sorption and solubility compared with both resin and self-adhesive materials.

## 1. Introduction

Dental luting cements are viscous pastes that acts on the interface between the indirect restoration and tooth surface to create an adhesive force through a chemical reaction [1]. An ideal luting material demonstrates standard properties including biocompatibility with oral tissues, adhesion to enamel or dentin [2], load-withstanding capacity, leakage resistance with a marginal seal, insolubility and resistance to water sorption [3].

Multiple factors in the oral environment including masticatory and parafunctional stresses, salivary contamination, pH variability, and temperature variances hinder the properties of luting agents [4]. As per the guidelines of the American Dental Association (ADA), water sorption and solubility are two essential criteria for evaluating luting cements. Evidence shows that luting agents undergo hydrolytic breakdown and dissolution of weak polymer chain bonds (Van der Waals force) when exposed to water or saliva [5,6,7]. This process reduces the physical and chemical properties of resin by plasticizing the polymer, leading to decreased bond strength and compromised mechanical properties [8,9]. Although water penetration helps adjust for shrinking stresses, it comes at a cost of reduced mechanical strength of the restoration [10,11]

Currently, available cements possesses some but not all desired properties of an ideal material. For instance, a cement should have low film thickness, long working time, controlled setting, with low solubility and microleakage. Among different cements, zinc phosphate (ZP), resin-modified glass ionomer (RMGI) and resin composites have low cement film thickness [8,12]. In addition, resin-based cement and glass ionomer cement (GIC) show comparatively longer working times compared to ZP and RMGI [13,14]. Resin cements have improved fracture toughness with better flexural modulus with low reactivity with water. Moreover, it is found thermal expansion and modulus of elasticity of GIC is similar to that of the root structure. Furthermore, solubility and microleakage is low among resin luting cements and GIC, in contrast to ZP and RMGI [13,14]. Regarding polymeric cements they have low solubility to fluids with high compressive strength. Moreover, they have double tensile strength compared to glass ionomer and zinc phosphate cements. Multiple factors influence variations and behavior of luting agents in the aqueous environment including the type of hydrophilic chemical species, nature of filler content, concentration and characteristics, mean particle size, coupling agents, and type of solvent [15,16].

Following ISO Specification No. 4049 water sorption and solubility of resin, cement material can be assessed over seven days. In a study by Mese et al., water sorption and solubility of resin-based and RMGI cement were assessed for a maximum duration of 5 weeks. From the study, it was concluded that RMGI showed more solubility and sorption compared to conventional resin cement [17]. In a further study by Marghalani, self-adhesive cements were compared for sorption and cement dissolution in water for 7 days. They reported that each cement showed different water sorption and solubility levels, with Rely X Unicem and GC-Cem exhibiting the lowest and highest sorption and solubility levels, respectively [17].

According to our knowledge of the indexed literature, the water sorption and solubility behavior of polymeric luting materials were assessed for a limited duration (5 weeks), however, water and salivary uptake in the oral cavity occurs well beyond this time frame [18,19,20]. Reports comparing the solubility and sorption of resin-based, resin-modified glass ionomer and self-etch cements for up to 180 days is lacking. It is hypothesized that those cements based on more hydrophilic monomers (self-etching self-adhesive resin cement and resin-modified glass-ionomer cement) will show greater solubility and water-sorption compared to resin cement. Therefore, this study aimed to evaluate the water-solubility and water-sorption characteristics of nine different polymeric luting agents over a 180-day water storage period.

## 2. Materials and Methods

Nine different cement materials were tested [Panavia F (PF), Rely X ARC (RA), Rely X Unicem (RU), Rely X luting plus (RL), Breez (BZ), Maxcem Elite (MX), BisCem (BC), FujiCem (FC) and FujiPlus (FP)] in the study (see Table 1 for the respective suppliers). Resin-based cements including Panavia F (Kuraray Medical, Tokyo, Japan) and Rely X ARC (3M ESPE, Seefeld, Germany) were used as controls (Table 1).

### 2.1. Specimen Preparation

Discs were prepared using molds made of natural acetal (Delrin) with internal dimensions of 15 mm ± 0.1 mm in diameter and 1.0 ± 0.1 mm depth. The molds were overfilled with the cements covered with a Mylar sheet and excess was removed with a glass slide. (Table 2) Each specimen was examined with the naked eye against a light to check for internal porosities. The light-cured samples were examined before and after light curing. A light-curing unit (L.E. Demetron I, Kerr Corporation, Orange, CA, USA) of 790 mW/cm^2^ was used to photo-activate the dual cured cements. Curing was done using a 13 mm tip with each specimen cured in overlapping sections, each for 40 s on both upper and lower surfaces (total of eight curing’s). Specimens were transferred to an oven, and maintained at 37 °C ± 1 °C. The self-cured specimens were placed in the oven for 60 min while the light and the dual-cured specimens were placed for 15 min. Specimen discs were randomly assigned to each of the four storage periods (7, 30, 90 and 180 days) in water (*n* = 52, 13 for each storage time, for each material).

### 2.2. Sorption and Solubility Analysis

A microbalance (AG285, Metter Toledo, Greifensee, Switzerland) with a precision of 0.01/mg was used for weighing the specimens. The water sorption/solubility test was performed according to the ISO 4049 (2000) specification for resin-based restorative materials. The methodology for assessment of water sorption and solubility was adopted from previous study [11]. Each specimen was finished by holding the periphery against 1000-grit abrasive paper on a non-rotating grinding table. Specimens were rotated so that the periphery was abraded, assuring the removal of flash. A visual inspection of the periphery ensured smoothness. Following grinding, specimens were transferred to a desiccator maintained at 37 °C ± 1 °C.

After 22 h, each specimen was removed and stored in a second desiccator maintained at 23 °C ± 1 °C for 2 h and then weighed. This cycle was repeated until the weight loss of each specimen was not more than 0.1 mg in any 24-h period; this constant weight was W_0_ (Baseline weight). After final drying, the mean diameter was determined by calculating the mean of two measurements at right angle to each other across the specimen surface. The mean thickness (mean of two measurements at 180°) was measured using a digital caliper with a precision of 0.01 mm (Max-cal, Cole Parmer Instrument Co., Chicago, IL, USA). The area and then the volume (V) in cubic millimeters were calculated. Before immersing the specimens in water, the specimen densities were determined from weight and volume measurements using the following equation:

ρ = W_0_/V (g/mm^3^)

Any samples with a density value less than 10 percent of the average were discarded due to the possible presence of internal voids. The specimens were immersed in 10 mL water (each specimen) for the selected storage period of 7, 30, 90 and 180 days with weekly water replacement. The specimens immersed for the one-week period were not subjected to a water change. Water pH was measured each time (pH = 5.5 ± 0.45) and after each period, each specimen was removed, washed with water, blot dried and air dried for 15 s. The weight for each specimen was measured to 0.1-mg accuracy within one minute of water removal. The weight measured after removal from the water storage was W_1_. Specimens were placed in a desiccator with freshly dried silica gel at an elevated temperature (90 °C) and then weighed at an equal interval until a constant weight was reached; this weight was the final weight (W_2_). Weight gains were measured by subtracting the original sample weight from the post-storage weight by the equation: W_1_ − W_2_. Water sorption percent (WSP %) and water solubility percent (WSL %) were calculated by the following equations:

WSP (%) = (W_1_ − W_2_) × 100/W_0_

WSL (%) = (W_0_ − W_2_) × 100/W_0_

### 2.3. Statistical Analysis

A full factorial two-way analysis of variance (ANOVA) model was used to assess the effect of luting agent and time period on water sorption and solubility. Pair-wise comparisons were adjusted using Tukey’s multiple comparison procedure. A significance level of 0.05 was used for all statistical tests.

## 3. Results

### 3.1. Water Sorption

Overall a significant difference in water sorption among the nine luting materials (*p* < 0.001), and the four testing periods 7, 30, 90 and 180 days (*p* < 0.001) were observed. Significant interaction was perceived between luting materials and time durations (*p* < 0.001) (Table 3). Table 3 presents the significant influence of cement type (trt), water immersion duration (period) and their combined effect on WSP.

There was no significant difference between water sorption results observed over the periods of seven days and 30 days (*p* > 0.05). The water sorption results significantly increased (*p* < 0.05) with increasing storage time (90, 180 days) with no significant difference between the storage periods of 90 and 180 days (*p* > 0.05) (Table 4).

Resin-modified glass-ionomers showed the highest percentage of water sorption of all the luting materials tested. RL had the highest water sorption among all cements (17.9% over 180 days), followed by FC (16.97% over 180 days) and FP (14.14% over 180 days), with significant differences between each one of them (*p* < 0.05). Resin-based luting agents showed the lowest percentage of water sorption. RA had the lowest water sorption (1.8% over 180 days) followed by PF (2.7% over 180 days) (*p* < 0.05) (Table 4). The self-adhesive luting materials showed a varied range of water sorption results. Among self-adhesive cements, RU had the lowest water sorption percentage (3.95% over 180 days) and BC had the highest (6.59% over 180 days) with a significant difference between them (*p* < 0.05) (Table 4).

Table 4 presents the statistical comparison of WSP based on immersion duration within the same cement type. Intragroup comparison in cements, BC, BZ, MX, PF and RA showed comparable outcomes (*p* > 0.01). Among FC, FP and RU, WSP significantly increased from 7 and 30 days to 90 and 180 days (*p* < 0.01) (Table 5). Table 5 presents statistical comparison of WSP among cement types within the same immersion duration subgroup. Majority of the comparison in Table 5 suggest a significant difference of WSP among cement types in a specific immersion duration subgroup.

**Figure 1:**
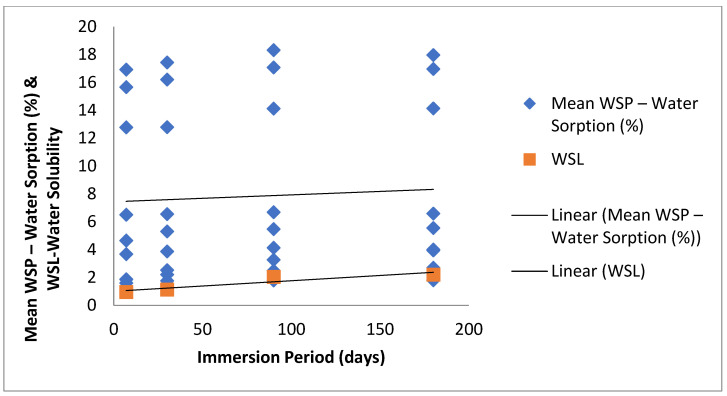
Trends in Water-Sorption (%) and Water-Solubility (%) among material for study duration (days).

There was significant interaction between the luting materials and time. RA, PF, BC, BZ and MX showed no significant change in their sorption with time changes resulting in a plot close to a straight line (Figure 1). By contrast, RU, RL, FC and FP showed significant increase in their sorption comparing 7-day storage and 90-day storage duration. Following 90-day water storage the increase in sorption was not significant up to 180 days (Figure 1).

### 3.2. Water Solubility (WSL)

Overall there was a significant difference in water solubility among the nine luting materials (*p* < 0.001) and four testing periods 7, 30, 90 and 180 days (*p* < 0.001). There was significant interaction between luting materials and time (*p* < 0.001) (Table 6). Table 3 presents the significant influence of cement type (trt), water immersion duration (period) and their combined effect on WSL.

RU, followed by the resin-based luting materials, displayed the lowest water solubility result (RU at 0.22% at 180 days; RL at 4.45% at 180 days). RA showed significantly lower water solubility (0.23% at 180 days) than PF (0.85% at 180 days) (Table 6). In addition, the resin-modified glass-ionomer luting materials exhibited the higher water solubility results compared to conventional resin cements (Table 7). Moreover, FC had the highest solubility (7.59% at 180 days), followed by FP (3.11% at 180 days), with significant differences among these luting materials (Table 7). The self-adhesive materials, excluding RU, showed lower solubility compared with the resin-based luting agents. However, RU exhibited higher water solubility than the resin-based luting materials. Among self-adhesive materials, water solubility was significantly different, where MX showed the highest water solubility percentage (1.55% at 180 days) followed by BC (1.12% at 180 days) and Breeze (1.15% at 180 days) respectively.

Table 8 presents the statistical comparison of WSL, based on immersion duration within the same cement type. Intragroup comparison in cements, RU, PF and BZ, showed comparable outcomes (*p* > 0.01). Among FC, FP and RL, WSL significantly increased from 7 days to 90 and 180 days respectively (*p* < 0.01) (Table 8). Table 9 presents statistical comparison of WSL among cement types within the same immersion duration subgroup. Majority of the comparison in Table 9 suggest a significant difference of WSL among cement types in a specific immersion duration subgroup. WSL for cements BC and BZ in 7 and 30 immersion duration subgroups showed comparable (*p* > 0.01) outcomes to cements MX, PF and RA respectively. Overall, increasing immersion duration showed significant increase in WSL among the tested materials (Table 10).

## 4. Discussion

The present study was based on the hypothesis that cements based on hydrophilic monomers (self-etching self-adhesive resin cement and resin-modified glass-ionomer cement) will show greater solubility and water-sorption compared to conventional cement. However, it was observed that most of the self-adhesive and RMGI luting agents showed higher WSL and WSP compared to resin luting agents except Rely-X Unicem. Therefore, the hypothesis was only partially accepted. Water solubility and sorption of luting material remain critical factors in the long-term survival of a restoration. Water can both decrease mechanical properties and degrade bonds between luting agents and tooth surface [8,18,19]

In the current study Fuji I GIC was selected as a negative control, however, it was not tested because it showed fractures and chipping before the first desiccating procedure. It is possible that Fuji I could not sustain desiccation due to the thickness of the discs prepared [21,22].The present study demonstrated that resin-modified glass ionomer may be be due to a multi-phase structure formation during the setting of RMGI featuring hydroxyethylmethacrylate (HEMA), an ionic component [23,24]. The hydrophilic and ionic components are considered as the sites where water uptake and retention take place in RMGI. HEMA, a significant monomer component of resin-modified glass ionomer is the main reason for hydroscopic expansion. HEMA in RMGI is found to be beneficial to compensate for setting shrinkage and resultant stresses [25,26]. However, the hygroscopic expansion accounts for the cracks observed in restorations after water immersion. In clinical settings, such hygroscopic expansion would be a reason for dental stress and postoperative sensitivity [23].

Amongst cement FC RMGI displayed the highest water sorption percentage. The differences may be attributed to the varying setting reaction FC is developed from the simplest resin-modified GI and replaces some of its water with polyacrylic acid (30–40%) [27,28]. FC undergoes a two-phase setting, the slow acid-base chemical reaction seen in conventional GIC preparations, and a photo-initiated co-polymerization reaction of HEMA-based methacrylate group [16,29]. On the other hand, FP cement, by contrast, relies on an additional third polymerization initiation through free radicals of its polymeric liquid. This linkage provides for more interlinking of the polymeric chains, resulting in less water sorption. Although the resin-modified glass-ionomers showed the highest percentage of water sorption and solubility of all the materials tested in the study, they have better WSL and WSP compared with other conventional luting materials such as zinc phosphate and zinc polycarboxylate as shown in previous literature [29]. In light of these findings, the use of such cement for cementation remains questionable. Taken together with the results of this study, it would seem to indicate that they may not be a good choice of material.

The self-adhesive luting materials (BZ, BC, MX) except RU, exhibited higher sorption percentages compared to conventional resin-based luting materials (PF and RA). While these materials demonstrate better wettability and penetration in the tooth structures due to their initial hydrophilic nature, they become more hydrophobic as the setting commences, thus displaying inferior hydrolytic stability [27]. Accurate information for filler content of self-adhesive luting materials is not available, hence the role of fillers in the behavior of the materials remains to be explored. However, in the resin matrix composition of these materials, it was found that urethane dimethacrylate (UDMA) polymers demonstrated more water uptake than the polymer-based non-hydroxylated bis-GMA analogs [29,30,31] UDMA, despite the very strong intermolecular interaction and rigid backbone, exhibited a low degree of conversion and is prone to water uptake. Moreover, in MX and BZ self-adhesive cement, HEMA and GDM have one of the highest hydrophilicities [32,33] HEMA has been shown to induce water sorption, leading to the expansion of the polymer matrix. HEMA showed more water-sorption compared to BisGMA polymers. Acidic monomer 4-MET in Breeze and HEMA, and GDM and tetramethyl butyl hydroperoxide in Maxcem may play a contributing role in water uptake and hygroscopic expansion [19,20]. No relation could be established between water-sorption behavior and water solubility, neither they indicate influence over each other. PF and RA, as a resin cement, contains 10-methacryloyloxydecyl dihydrogen phosphate (10-MDP) as the etching monomer. The long carbonyl chain of 10-MDP makes this monomer quite hydrophobic contributing to low water sorption over time [20].

The existing study compared WSP and WSL among different contemporary luting agents, however other properties including their long-term mechanical properties and durability are critical for clinical performance. The present study assessed WSL and WSP in an in-vitro setting. In a study by Gemalmaz et al., the solubility of glass-ionomers was assessed by bonding the samples to the flanges of a denture and letting the patients wear the prosthesis for a period of time [4]. Within the limitations of the present study, similar methodologies investigating solubility and sorption behavior of luting agents bonded to tooth structure and restorative material in-vivo with correlations to their mechanical performances are recommended. Contraction stress, water absorbency, magnitude of reduction, and dynamics of stress of luting materials needs further evaluation and investigation. In clinical conditions, water absorption may help in the closure of contraction gaps around composite filling materials. It is worth emphasizing that the absorption can, in some cases, result in significant hygroscopic expansion and, thus, be damaging to the resin material and bonded tooth structure

## 5. Conclusions

Resin-based luting materials had the lowest sorption and solubility. Rely X Unicem self-adhesive luting materials were comparable to resin luting materials for WSL and WSP. Resin-modified glass-ionomer showed the highest water sorption and solubility compared with both resin and self-adhesive materials.

## Figures and Tables

**Table 1:** List of luting agents assessed in the study.

Luting Materials	Type	Composition	Manufacturer
Panavia F (PF)	Conventional resin Cement	Base: 10-MDP, 5-NMSA, silica, dimethacrylates, initiator. Catalyst: barium glass, sodium fluoride, dimethacrylates, BPO	Kuraray Medical, Tokyo, Japan
Rely X ARC (RA)	Conventional resin Cement	Past A: BisGMA, TEGDMA, zirconia/silica filler 67.5% wt, dimethacrylate monomer. Past B: contain peroxide.	3M ESPE, Seefeld, Germany
Rely X Unicem (RU)	Self-adhesive resin cement	Self-etch cement powder: glass powder, silica, calcium hydroxide, pigment, substitude pyrimidine and peroxy compound. (Filler load 72% wt, particles size < 9.5 µm) Liquid initiator: methacrylated phosphoric, dimethacrylate, acetate, stabilizer and initiator.	3M ESPE, Seefeld, Germany
Rely X luting plus (RL)	Resin Modified Glass Ionomer Cement	Past A: fluoroaluminosilicate glass, proprietary reducing agent, HEMA, water, opacity. Past B: metharylate polycarboxylic acid, BisGMA, HEMA, water, potassium persulfate, zirconia silica fillers.	3M ESPE, Seefeld, Germany
Breez (BZ)	Self-adhesive resin cement	BisGMA, UDMA, TEGDMA, HEMA, 4-MET, barium glass, silica, BiOcl, Ca, Al, F	Pentron, Wallingford, CT, USA
Maxcem Elite (MX)	Self-adhesive resin cement	Glyceroldimethacrylate dihydrogrn phosphate (GPDM), mono-, di-, tri-functional methacrylate monomers, self-cured redox initiator, photoinitiator (camphorquinone), stabilizer. (67% wt fillers, filler size 3.6 µm): barium glass, fluoroaluminosilicate and silica.	Kerr, Orange, CA, USA
BisCem (BC)	Self-adhesive resin cement	Bis (Hydroxyethylmethacrylate) 10–30%, Phosphate (base) 40–70%, Tetraethylene glycol, dimethacrylate, dental glass.	BISCO, Chicago, IL, USA
FujiCem (FC)	Resin Modified Glass Ionomer Cement	(% Chemical components by WT and exposure limits) Distilled water30–40%, Polyacrylic acid 30–40%, Benzenesulfonic acid sodium salt 2–3%, Silica powder 2%.	GC America, Chicago IL, USA
Fuji Plus (FP)	Resin Modified Glass Ionomer Cement	Powder: silica glass, Liquid: polyacrylic acid, 2-hydroxyethil methacrylate, di-2-methacryloxethy-2, 2,4, trimethyl hexamethylene dicarbamate, tartaric acid.	GC America, Chicago IL, USA

**Table 2:** Manufacturers’ instructions for the cements assessed in the study.

Materials	Manufacturers’ Instructions
Panavia F (PF) (Kuraray Medical, Tokyo, Japan)	1. Dispense Equal amount of paste A and paste B. 2. Mix sufficient paste A and paste B on the mixing plate for 20 s. 3. Light cure for 20 s.
Fuji-Plus (FP) (GC America, Chicago, IL, USA)	1. Powder and liquid dispensing: powder to liquid ratio is 2.0 g/1.0 g. (1 level large scoop of powder to 3 drops of liquid. 2. Mixing: Dispense powder and liquid onto the pad. Using the plastic spatula, add all the powder to the liquid. Mix rapidly for 20 s. 3. Maintain isolation until set is verified (Approx. 4 min).
FujiCem (FC) (GC America, Chicago, IL, USA)	- Cement supplied as a paste pack. 1. Insert Paste Pak into dispenser and twist into position. 2. Dispense desired amount of material. 3. Mix with spatula for 10 s. 4. Cement set in 3 min, after remove the excess.
Rely X ARC (RA) (3M ESPE, Seefeld, Germany)	1. Apply and evenly distribute a thin layer of cement to the bonding surface of the indirect restoration. 2. Setting time 3–5 min. 3. Light cure for 40 s or allowed to self-cure for 10 min from start of mix.
Rely X Unicem (RU) (3M ESPE, Seefeld, Germany)	1. Mix the 3M™ESPE™RelyX™Unicem Self-Adhesive Universal Resin Cement capsule in a high-frequency mixing unit (e.g., Capmix™) for 15 s or in the Rotomix™capsule-mixing unit for 10 s (see also the section on “Times”). 2. Application: Insert the capsule in the Aplicap Applier after mixing and open the nozzle as far as possible. Protect the working area from water and saliva during application. Working time from the start of mixing 2 min. 3. Light curing: 20 s for each surface. 4. Self-curing: set time after start of mixing 5 min.
Rely X Luting Plus (RL) (3M ESPE, Seefeld, Germany)	1. Mixing: Using a cement spatula, mix the powder into the liquid. To minimize water evaporation and maximize working time, continue spatulation of the powder and liquid to a small area of the mixing pad. All of the powder should be incorporated into the liquid within 30 s. 2. Working Time of the standard powder/liquid ratio is at least 2.5 min from the start of mix at a room temperature of 73 °F (23 °C).
Breez (BZ) (Pentron, Wallingford, CT, USA)	1. Dispense: Dispense Breeze™Cement directly into restoration. 2. Place: Seat restoration. 3. Cure: Light cure or self-cure.
Maxcem Elite (MX) (Kerr, Orange, CA, USA)	1. Dispensing the material 2. Allow Maxcem to sit undisturbed for 1 1/2 min before light curing. 3. Light-cure all surfaces including margins for 20 s.
BisCem (BC) (BISCO, Chicago, IL, USA)	1. Cement is supplied in a single syringe. 2. Fill restoration with BisCem. 3. Seat the restoration. 4. Light cure for 3–5 s, to aid in cement removal. 5. Excess cement is then easily removed. 6. Light cure for 20–30 s.

**Table 3:** Showing significant influence of cement type and time duration on water-sorption of the cements tested (ANOVA).

Source	DF	Type III SS	Mean Square	F Value	Pr > F
trt	8	15,786.94	1973.36	5674.98	<0.0001
period	3	62.87	20.95	60.27	<0.0001
trt*period	24	36.43	1.51	4.37	<0.0001

trt: cement type, period: immersion duration.

**Table 4:** Statistical intra group comparison (cement) of WSP (%) among different immersion sub-groups (Pair-wise comparisons within luting agent; Tukey adjusted *p*-values).

Cement Type	Immersion Duration	Mean (SD)	Period	7	30	90	180
BC	7	6.51 (0.28)	7		1.0000	1.0000	1.0000
BC	30	6.55 (0.21)	30	1.0000		1.0000	1.0000
BC	90	6.69 (0.27)	90	1.0000	1.0000		1.0000
BC	180	6.59 (0.17)	180	1.0000	1.0000	1.0000	
BZ	7	4.66 (0.18)	7		0.6276	0.1846	0.0533
BZ	30	5.31 (0.26)	30	0.6276		1.0000	1.0000
BZ	90	5.47 (0.17)	90	0.1846	1.0000		1.0000
BZ	180	5.55 (0.17)	180	0.0533	1.0000	1.0000	
FC	7	15.67 (0.55)	7		0.9205	<0.0001	<0.0001
FC	30	16.21 (0.47)	30	0.9205		0.1197	0.2991
FC	90	17.08 (0.58)	90	<0.0001	0.1197		1.0000
FC	180	16.97 (0.67)	180	<0.0001	0.2991	1.0000	
FP	7	12.78 (1.24)	7		1.0000	<0.0001	<0.0001
FP	30	12.79 (1.02)	30	1.0000		<0.0001	<0.0001
FP	90	14.12 (1.99)	90	<0.0001	<0.0001		1.0000
FP	180	14.14 (1.53)	180	<0.0001	<0.0001	1.0000	
MX	7	3.68 (0.21)	7		1.0000	0.9990	1.0000
MX	30	3.88 (0.19)	30	1.0000		1.0000	1.0000
MX	90	4.14 (0.16)	90	0.9990	1.0000		1.0000
MX	180	4.01 (0.25)	180	1.0000	1.0000	1.0000	
PF	7	1.60 (0.21)	7		0.8105	0.0767	0.0025
PF	30	2.21 (0.17)	30	0.8105		1.0000	0.9786
PF	90	2.49 (0.21)	90	0.0767	1.0000		1.0000
PF	180	2.70 (0.18)	180	0.0025	0.9786	1.0000	
RA	7	1.87 (0.19)	7		1.0000	1.0000	1.0000
RA	30	1.76 (0.08)	30	1.0000		1.0000	1.0000
RA	90	1.79 (0.06)	90	1.0000	1.0000		1.0000
RA	180	1.80 (0.08)	180	1.0000	1.0000	1.0000	
RL	7	16.92 (0.46)	7		0.9603	<0.0001	0.0058
RL	30	17.44 (0.6)	30	0.9603		0.0811	0.9683
RL	90	18.32 (0.34)	90	<0.0001	0.0811		1.0000
RL	180	17.97 (0.8)	180	0.0058	0.9683	1.0000	
RU	7	1.86 (0.11)	7		0.6757	<0.0001	<0.0001
RU	30	2.53 (0.21)	30	0.6757		0.3584	<0.0001
RU	90	3.27 (0.35)	90	<0.0001	0.3584		0.6029
RU	180	3.95 (0.42)	180	<0.0001	<0.0001	0.6029	

PF = Panavia F; RA = Rely X ARC; RU = Rely X Unicem; RL = Rely X Luting Plus; BZ = Breez; MX = Maxcem Elite; BC = BisCem; FC = FujiCem; FP = Fuji Plus.

**Table 5:** Statistical comparison of WSP (%) among different cement types in a specific immersion duration subgroup. (Pair-wise comparisons within immersion duration; Tukey adjusted *p*-values).

ID	CT	Mean	i/j	BC	BZ	FC	FP	MX	PF	RA	RL	RU
7	BC	6.51	BC		<0.0001	<0.0001	<0.0001	<0.0001	<0.0001	<0.0001	<0.0001	<0.0001
7	BZ	4.66	BZ	<0.0001		<0.0001	<0.0001	0.0136	<0.0001	<0.0001	<0.0001	<0.0001
7	FC	15.67	FC	<0.0001	<0.0001		<0.0001	<0.0001	<0.0001	<0.0001	<0.0001	<0.0001
7	FP	12.78	FP	<0.0001	<0.0001	<0.0001		<0.0001	<0.0001	<0.0001	<0.0001	<0.0001
7	MX	3.68	MX	<0.0001	0.0136	<0.0001	<0.0001		<0.0001	<0.0001	<0.0001	<0.0001
7	PF	1.60	PF	<0.0001	<0.0001	<0.0001	<0.0001	<0.0001		1.0000	<0.0001	1.0000
7	RA	1.87	RA	<0.0001	<0.0001	<0.0001	<0.0001	<0.0001	1.0000		<0.0001	1.0000
7	RL	16.92	RL	<0.0001	<0.0001	<0.0001	<0.0001	<0.0001	<0.0001	<0.0001		<0.0001
7	RU	1.86	RU	<0.0001	<0.0001	<0.0001	<0.0001	<0.0001	1.0000	1.0000	<0.0001	
30	BC	6.55	BC		<0.0001	<0.0001	<0.0001	<0.0001	<0.0001	<0.0001	<0.0001	<0.0001
30	BZ	5.31	BZ	<0.0001		<0.0001	<0.0001	<0.0001	<0.0001	<0.0001	<0.0001	<0.0001
30	FC	16.21	FC	<0.0001	<0.0001		<0.0001	<0.0001	<0.0001	<0.0001	0.0002	<0.0001
30	FP	12.79	FP	<0.0001	<0.0001	<0.0001		<0.0001	<0.0001	<0.0001	<0.0001	<0.0001
30	MX	3.88	MX	<0.0001	<0.0001	<0.0001	<0.0001		<0.0001	<0.0001	<0.0001	<0.0001
30	PF	2.21	PF	<0.0001	<0.0001	<0.0001	<0.0001	<0.0001		0.9948	<0.0001	1.0000
30	RA	1.76	RA	<0.0001	<0.0001	<0.0001	<0.0001	<0.0001	0.9948		<0.0001	0.2593
30	RL	17.44	RL	<0.0001	<0.0001	0.0002	<0.0001	<0.0001	<0.0001	<0.0001		<0.0001
30	RU	2.53	RU	<0.0001	<0.0001	<0.0001	<0.0001	<0.0001	1.0000	0.2593	<0.0001	
90	BC	6.69	BC		0.0002	<0.0001	<0.0001	<0.0001	<0.0001	<0.0001	<0.0001	<0.0001
90	BZ	5.47	BZ	0.0002		<0.0001	<0.0001	0.0007	<0.0001	<0.0001	<0.0001	<0.0001
90	FC	17.08	FC	<0.0001	<0.0001		<0.0001	<0.0001	<0.0001	<0.0001	0.0002	<0.0001
90	FP	14.12	FP	<0.0001	<0.0001	<0.0001		<0.0001	<0.0001	<0.0001	<0.0001	<0.0001
90	MX	4.14	MX	<0.0001	0.0007	<0.0001	<0.0001		<0.0001	<0.0001	<0.0001	0.2814
90	PF	2.49	PF	<0.0001	<0.0001	<0.0001	<0.0001	<0.0001		0.4539	<0.0001	0.2485
90	RA	1.79	RA	<0.0001	<0.0001	<0.0001	<0.0001	<0.0001	0.4539		<0.0001	<0.0001
90	RL	18.32	RL	<0.0001	<0.0001	0.0002	<0.0001	<0.0001	<0.0001	<0.0001		<0.0001
90	RU	3.27	RU	<0.0001	<0.0001	<0.0001	<0.0001	0.2814	0.2485	<0.0001	<0.0001	
180	BC	6.59	BC		0.0041	<0.0001	<0.0001	<0.0001	<0.0001	<0.0001	<0.0001	<0.0001
180	BZ	5.55	BZ	0.0041		<0.0001	<0.0001	<0.0001	<0.0001	<0.0001	<0.0001	<0.0001
180	FC	16.97	FC	<0.0001	<0.0001		<0.0001	<0.0001	<0.0001	<0.0001	0.0189	<0.0001
180	FP	14.14	FP	<0.0001	<0.0001	<0.0001		<0.0001	<0.0001	<0.0001	<0.0001	<0.0001
180	MX	4.01	MX	<0.0001	<0.0001	<0.0001	<0.0001		<0.0001	<0.0001	<0.0001	1.0000
180	PF	2.70	PF	<0.0001	<0.0001	<0.0001	<0.0001	<0.0001		0.0438	<0.0001	0.0001
180	RA	1.80	RA	<0.0001	<0.0001	<0.0001	<0.0001	<0.0001	0.0438		<0.0001	<0.0001
180	RL	17.97	RL	<0.0001	<0.0001	0.0189	<0.0001	<0.0001	<0.0001	<0.0001		<0.0001
180	RU	3.95	RU	<0.0001	<0.0001	<0.0001	<0.0001	1.0000	0.0001	<0.0001	<0.0001	

ID, Immersion duration, CT, Cement type.

**Table 6:** Showing significant influence of cement type and time duration on water-solubility (WSL %) of the cements tested (ANOVA).

Source	DF	Type III SS	Mean Square	F Value	Pr > F
trt	8	966.47	120.80	1999.53	<0.0001
period	3	142.91	47.63	788.48	<0.0001
trt*period	24	236.21	9.84	162.9	<0.0001

trt: cement type, period: immersion duration.

**Table 7:** Comparison of overall WSL (%) means among cements investigated in the study using Tukey Multiple comparisons test.

Study Groups	Mean
FC	4.83 ^A^
RL	3.25 ^B^
FP	1.99 ^C^
MX	1.11 ^D^
BC	0.94 ^E^
BZ	0.93 ^E^
PF	0.67 ^F^
RA	0.46 ^G^
RU	0.13 ^H^

Means with the same superscript letter are not significantly different.

**Table 8:** Statistical intra group comparison (cement) of WSL (%) among different immersion sub-groups (Pair-wise comparisons within luting agent; Tukey adjusted *p*-values).

Cement Type	Immersion Duration	Mean	i/j	7	30	90	180
BC	7	0.62	7		1.0000	<0.0001	0.0005
BC	30	0.73	30	1.0000		<0.0001	0.0356
BC	90	1.27	90	<0.0001	<0.0001		0.9999
BC	180	1.12	180	0.0005	0.0356	0.9999	
BZ	7	0.75	7		1.0000	0.8547	0.0194
BZ	30	0.83	30	1.0000		0.9996	0.2593
BZ	90	0.99	90	0.8547	0.9996		0.9999
BZ	180	1.15	180	0.0194	0.2593	0.9999	
FC	7	2.29	7		<0.0001	<0.0001	<0.0001
FC	30	3.24	30	<0.0001		<0.0001	<0.0001
FC	90	6.72	90	<0.0001	<0.0001		<0.0001
FC	180	7.59	180	<0.0001	<0.0001	<0.0001	
FP	7	0.97	7		1.0000	<0.0001	<0.0001
FP	30	1.07	30	1.0000		<0.0001	<0.0001
FP	90	2.78	90	<0.0001	<0.0001		0.2338
FP	180	3.11	180	<0.0001	<0.0001	0.2338	
MX	7	0.75	7		0.4821	0.0844	<0.0001
MX	30	1.04	30	0.4821		1.0000	0.0002
MX	90	1.16	90	0.0844	1.0000		0.1569
MX	180	1.55	180	<0.0001	0.0002	0.1569	
PF	7	0.42	7		1.0000	0.0227	0.0076
PF	30	0.56	30	1.0000		0.7204	0.4845
PF	90	0.82	90	0.0227	0.7204		1.0000
PF	180	0.85	180	0.0076	0.4845	1.0000	
RA	7	0.74	7		0.9907	0.0051	0.0001
RA	30	0.55	30	0.9907		0.8676	0.2773
RA	90	0.31	90	0.0051	0.8676		1.0000
RA	180	0.23	180	0.0001	0.2773	1.0000	
RL	7	1.88	7		0.0230	<0.0001	<0.0001
RL	30	2.28	30	0.0230		<0.0001	<0.0001
RL	90	4.42	90	<0.0001	<0.0001		1.0000
RL	180	4.45	180	<0.0001	<0.0001	1.0000	
RU	7	0.05	7		1.0000	1.0000	0.9998
RU	30	0.07	30	1.0000		1.0000	0.9999
RU	90	0.16	90	1.0000	1.0000		1.0000
RU	180	0.22	180	0.9998	0.9999	1.0000	

**Table 9:** Statistical comparison of WSL (%) among different cement types in a specific immersion duration subgroups. (Pair-wise comparisons within immersion duration; Tukey adjusted *p*-values).

ID	CT	Mean	i/j	BC	BZ	FC	FP	MX	PF	RA	RL	RU
7	BC	0.62	BC		1.0000	<0.0001	0.1642	1.0000	0.9883	1.0000	<0.0001	<0.0001
7	BZ	0.75	BZ	1.0000		<0.0001	0.9440	1.0000	0.2342	1.0000	<0.0001	<0.0001
7	FC	2.29	FC	<0.0001	<0.0001		<0.0001	<0.0001	<0.0001	<0.0001	0.0116	<0.0001
7	FP	0.97	FP	0.1642	0.9440	<0.0001		0.9511	<0.0001	0.9197	<0.0001	<0.0001
7	MX	0.75	MX	1.0000	1.0000	<0.0001	0.9511		0.2201	1.0000	<0.0001	<0.0001
7	PF	0.42	PF	0.9883	0.2342	<0.0001	<0.0001	0.2201		0.2777	<0.0001	0.1237
7	RA	0.74	RA	1.0000	1.0000	<0.0001	0.9197	1.0000	0.2777		<0.0001	<0.0001
7	RL	1.88	RL	<0.0001	<0.0001	0.0116	<0.0001	<0.0001	<0.0001	<0.0001		<0.0001
7	RU	0.05	RU	<0.0001	<0.0001	<0.0001	<0.0001	<0.0001	0.1237	<0.0001	<0.0001	
30	BC	0.73	BC		1.0000	<0.0001	0.1646	0.3417	0.9989	0.9953	<0.0001	<0.0001
30	BZ	0.83	BZ	1.0000		<0.0001	0.8624	0.9695	0.6663	0.5414	<0.0001	<0.0001
30	FC	3.24	FC	<0.0001	<0.0001		<0.0001	<0.0001	<0.0001	<0.0001	<0.0001	<0.0001
30	FP	1.07	FP	0.1646	0.8624	<0.0001		1.0000	0.0001	<0.0001	<0.0001	<0.0001
30	MX	1.04	MX	0.3417	0.9695	<0.0001	1.0000		0.0007	0.0003	<0.0001	<0.0001
30	PF	0.56	PF	0.9989	0.6663	<0.0001	0.0001	0.0007		1.0000	<0.0001	0.0003
30	RA	0.55	RA	0.9953	0.5414	<0.0001	<0.0001	0.0003	1.0000		<0.0001	0.0006
30	RL	2.28	RL	<0.0001	<0.0001	<0.0001	<0.0001	<0.0001	<0.0001	<0.0001		<0.0001
30	RU	0.07	RU	<0.0001	<0.0001	<0.0001	<0.0001	<0.0001	0.0003	0.0006	<0.0001	
90	BC	1.27	BC		0.6318	<0.0001	<0.0001	1.0000	0.0025	<0.0001	<0.0001	<0.0001
90	BZ	0.99	BZ	0.6318		<0.0001	<0.0001	1.0000	0.9992	<0.0001	<0.0001	<0.0001
90	FC	6.72	FC	<0.0001	<0.0001		<0.0001	<0.0001	<0.0001	<0.0001	<0.0001	<0.0001
90	FP	2.78	FP	<0.0001	<0.0001	<0.0001		<0.0001	<0.0001	<0.0001	<0.0001	<0.0001
90	MX	1.16	MX	1.0000	1.0000	<0.0001	<0.0001		0.4279	<0.0001	<0.0001	<0.0001
90	PF	0.82	PF	0.0025	0.9992	<0.0001	<0.0001	0.4279		0.0001	<0.0001	<0.0001
90	RA	0.31	RA	<0.0001	<0.0001	<0.0001	<0.0001	<0.0001	0.0001		<0.0001	1.0000
90	RL	4.42	RL	<0.0001	<0.0001	<0.0001	<0.0001	<0.0001	<0.0001	<0.0001		<0.0001
90	RU	0.16	RU	<0.0001	<0.0001	<0.0001	<0.0001	<0.0001	<0.0001	1.0000	<0.0001	
180	BC	1.12	BC		1.0000	<0.0001	<0.0001	0.0060	0.6771	<0.0001	<0.0001	<0.0001
180	BZ	1.15	BZ	1.0000		<0.0001	<0.0001	0.0208	0.4133	<0.0001	<0.0001	<0.0001
180	FC	7.59	FC	<0.0001	<0.0001		<0.0001	<0.0001	<0.0001	<0.0001	<0.0001	<0.0001
180	FP	3.11	FP	<0.0001	<0.0001	<0.0001		<0.0001	<0.0001	<0.0001	<0.0001	<0.0001
180	MX	1.55	MX	0.0060	0.0208	<0.0001	<0.0001		<0.0001	<0.0001	<0.0001	<0.0001
180	PF	0.85	PF	0.6771	0.4133	<0.0001	<0.0001	<0.0001		<0.0001	<0.0001	<0.0001
180	RA	0.23	RA	<0.0001	<0.0001	<0.0001	<0.0001	<0.0001	<0.0001		<0.0001	1.0000
180	RL	4.45	RL	<0.0001	<0.0001	<0.0001	<0.0001	<0.0001	<0.0001	<0.0001		<0.0001
180	RU	0.22	RU	<0.0001	<0.0001	<0.0001	<0.0001	<0.0001	<0.0001	1.0000	<0.0001	

ID, Immersion duration, CT, Cement type.

**Table 10:** Comparison of overall WSL (%) means among time periods investigated in the study using Tukey Multiple comparisons test.

Study Period	Mean
180	2.21 ^a^
90	2.05 ^b^
30	1.14 ^c^
7	0.96 ^d^

Means with the same superscript letter are not significantly different.

## Data Availability

The data presented in this study are available on request from the corresponding author.
